# Effect of Different Prebiotic Saccharides on *Listeria monocytogenes* Adherence to Human Adenocarcinoma Caco-2 Cell Line

**DOI:** 10.3390/cimb47110891

**Published:** 2025-10-28

**Authors:** Tereza Kodešová, Ivo Doskočil, Eva Vlková, Hana Šubrtová Salmonová

**Affiliations:** Department of Microbiology, Nutrition and Dietetics, Faculty of Agrobiology, Food and Natural Resources, Czech University of Life Science in Prague, Kamýcká 129, 165 21 Prague, Czech Republic; kodesovat@af.czu.cz (T.K.); doskocil@af.czu.cz (I.D.); vlkova@af.czu.cz (E.V.)

**Keywords:** *Listeria monocytogenes*, prebiotics, oligosaccharides, adherence, pathogen, beta-(1,3)-D-glucan

## Abstract

*Listeria monocytogenes* (LM) is one of the most emerging pathogens responsible for the serious foodborne disease listeriosis. The risk of disease outbreaks can be reduced by suppressing the adherence of LM to the intestinal epithelial cells. This effect can be achieved by prebiotic supplementation. The aim of this work was to determine the effect of prebiotics beta-(1,3)-D-glucan, inulin, fructooligosaccharides, galactooligosaccharides, lactulose, raffinose, stachyose, human milk oligosaccharides (HMOs), and 2’-fucosyllactose on the ability of LM to adhere to the human adenocarcinoma Caco-2 cell line. Despite strain-specific variability, a statistically significant reduction in LM adhesion to intestinal epithelial cells was observed in the presence of beta-(1,3)-D-glucan (~60% reduction), inulin (~46%), and HMOs (~44%). In contrast, the remaining tested prebiotics did not show a significant impact on LM adhesion. These findings highlight the potential of specific prebiotics, especially beta-glucans, to limit LM adherence, suggesting a protective effect for the host.

## 1. Introduction

*Listeria monocytogenes* (LM) is a foodborne pathogen responsible for the serious foodborne disease listeriosis, which is the fifth most reported zoonosis in the EU [1,2,3,4]. Despite the high prevalence of LM in the environment and its frequent contamination of food, the infections caused by this pathogen are relatively rare (around 2000 cases/year/EU), but they are characterized by a high mortality rate compared to other foodborne and zoonotic diseases. Approximately 10% of infected people in the EU succumb to the disease [1]. LM is subdivided into 13 known serotypes, among which 1/2a, 1/2b, 1/2c, and 4b are the most common and account for 95% of all listeriosis outbreaks. Listeriosis can occur in several forms, and disease severity depends on both the listeria strain and the host’s immune status. The infectious doses for healthy people may vary from 10^5^ to 10^9^ cells, and for high-risk individuals (children, the elderly, immunocompromised people, and pregnant women) this can be as low as 10^2^ to 10^3^ cells. A less serious form of listeriosis is the non-invasive disease, i.e., gut colonization, which might be asymptomatic or, in more severe cases, manifests as febrile gastroenteritis with symptoms such as diarrhea, nausea, and vomiting. Highly invasive strains of LM cause dangerous invasive systemic diseases such as sepsis, meningitis, or meningoencephalitides. In the worst cases, these bacteria cause death, and in pregnant women, miscarriage. The most invasive LM serotype is 4b, which is responsible for 50% of invasive listeriosis [2,3,5,6,7].

LM enters the gastrointestinal tract after consumption of contaminated food or by mother-to-child transmission (neonatal listeriosis). The virulence of LM depends on the specific virulent proteins, mostly occurring in the LM cell wall and cytoplasmic membrane. The initial step in establishing an LM infection is the adhesion of the organism to the intestinal epithelium of the host and subsequent penetration into intestinal epithelial cells. In this phase, multiple virulence proteins are involved; the most important are listeria adhesive protein (LAP), internalins (e.g., InA, InB, InC, InJ), and invasion-associated protein (iap) [3,6,8,9,10,11,12]. Then the pathogen continues to penetrate through the gastrointestinal tract (GIT) epithelium into the blood and lymph, and gradually spreads into the whole body where other virulence factors are expressed, e.g., listeriolysin O (LLO), phosphatidylinositol-specific phospholipase C (PlcA), broad-range phospholipase C (PlcB), actin assembly-inducing protein (ActA), metalloprotease (Mpl), etc. Most of these mechanisms are regulated by the positive regulatory factor A (PrfA) [3,8,10,13,14]. Invasion and infection of the LM host is therefore a very complex process. Further, by suppressing the adherence of LM to the intestinal epithelium, the risk of pathogen penetration into the host’s body and disease outbreaks is reduced [15,16,17].

In the mammalian gastrointestinal tract, multiple defense mechanisms exist that prevent LM growth, epithelial penetration, and systemic dissemination [18]. These mechanisms can be supported by, e.g., immune boosting or the promotion of gut microbiota. Both can be achieved through the consumption of prebiotics. Prebiotics are defined as substrates that are selectively utilized by host microorganisms conferring a health benefit to the host [19]. The consumption of prebiotics induces direct or indirect effects on the gut-associated epithelial and immune cells that leads to either pro- or anti-inflammatory responses [19,20,21,22,23]. One of the direct positive effects of prebiotics on intestinal epithelial cells is reduction in the adhesion of pathogens to intestinal epithelial cells, for example, by attenuating bacterial virulence or through directly sticking to the bacterial surface [24,25,26,27]. One of the main indirect effects, prebiotics also act as a substrate for the commensal gut bacteria that produce short-chain fatty acids (SCFAs) that modulate the immune response [28]. These mostly health-beneficial bacteria also produce antimicrobial secondary metabolites, e.g., bacteriocins, and competitively inhibit the attachment sites of intestinal cells to resist the invasion of pathogens. All these mechanisms reduce the ability of pathogens to adhere to the intestinal barrier and invade the host body [19,20,21,22,23,29,30,31,32].

This study was prompted by the limited scope of existing research investigating the impact of prebiotics on LM adhesion to intestinal epithelial cells, which has been limited to a narrow selection of prebiotic compounds (i.e., inulin, galactooligosaccharides, xylooligosaccharides, polydextrose [27], human milk oligosaccharides or mannanoligosaccharides [33]), often using varying experimental methodologies, making cross-study comparisons difficult. Moreover, interest in this topic increased following our previous study [34], which demonstrated that LM is capable of utilizing beta-1,3-D-glucan, a commonly used prebiotic, as a sole carbon source. This finding raised questions about the dual role of certain prebiotics as both potential growth substrates and modulators of pathogen behavior. The aim of this study was therefore to systematically investigate the influence of LM adherence to Caco-2 cell lines using the prebiotic saccharides, namely beta-(1,3)-D-glucan, inulin, fructooligosaccharides (FOSs), galactooligosaccharides (GOSs), lactulose, raffinose, stachyose, human milk oligosaccharides (HMOs), and 2’-fucosyllactose, used in a previous study by Kodešová et al. [34] by applying a unified methodology.

## 2. Materials and Methods

### 2.1. Tested Strains

*Listeria* strains were isolated from foodstuffs (n = 243) purchased on the market in the Czech Republic and from swabs (n = 8) taken from a work surface in a food processing plant according to the ISO 11290-1:2017 standard [35]. Presumptive *L. monocytogenes* colonies were identified and characterized as described in the study by Kodešová [34]. Briefly, isolates were preliminary identified at the genus level by the MALDI-TOF MS using the extraction method of ribosomal proteins. Confirmed strains of *Listeria* were identified by 16S rDNA sequencing [36]. Amplification was performed using primers fD1 [5′ AGAGTTTGATCCTGGCTCAG 3′] and rP2 [5′ ACGGCTACCTTGTTACGACTT 3′] [36]. Sequencing data were compared with sequences available in the GenBank nucleotide and databases in NCBI [37] and EZ Biocloud [38]. The sequences were deposited in GenBank via the BankIt program at the NCBI website (https://www.ncbi.nlm.nih.gov/WebSub/?tool=genbank), accessed on 29 October 2023. Serotype was determined with the slide agglutination method using commercial antisera according to the manufacturer’s instructions (Mast Group, Liverpool, UK). Haemolytic activity was explored in vitro by hemolysis testing on Columbia blood agar (Oxoid, Basingstoke, UK) supplemented with 5% sheep blood (*v*/*v*). Since LM adherence ability was the main focus of this study, the tested strains were further analyzed by PCR for the presence of genes linked to virulence factors involved in host cell adherence and invasion, such as listeria adhesive protein (LAP), internalin A (InlA), internalin B (InlB), internalin C (InlC), internalin J (InlJ), and invasion-associated protein (iap, p60) [27,39,40,41]. More details are provided in Table S1.

Out of the total number of samples, 18 foods and 3 swabs originated from different manufacturers were confirmed to be LM-positive. In total, 7 isolates of the 1/2a LM serotype, 4 of the 1/2b serotype, 4 of the 1/2c serotype, and 6 of the 4b serotype were obtained. Twelve strains, representing 4 of the most common serotypes (4b, 1/2a, 1/2b, and 1/2c) of different origin, that possess beta-hemolytic activity, were selected for testing. In all strains, all the above-mentioned virulence factors were detected. The list of LM strains is shown in Table 1.

### 2.2. Cell Culture

The human colorectal adenocarcinoma cell line Caco-2 (ATCC HTB-37) was used for testing. The cell line was seeded at a concentration of 2 × 10^5^ cells per well into cell culture a 96-well culture plate (VWR, CZ), and cultured in Dulbecco’s Modified Eagle’s Medium (DMEM) supplemented with 20% fetal bovine serum, 1% non-essential amino acids, 100 μg/mL penicillin, and 100 μg/mL streptomycin. All solutions were purchased from Biowest (Nuaillé, France). Cultures were incubated at 37 °C in a humidified atmosphere of 5% CO_2_ and 95% air (*v*/*v*) for 3 weeks. The medium was changed every two days, and the cells were subcultured at 80% confluence every week.

### 2.3. Prebiotic Saccharides and Testing Media Preparation

The most commonly used prebiotics for human and livestock nutrition were used in the form of food supplements: beta-(1,3)-D-glucan (Brainway Inc., Prague, Czech Republic), inulin (Frutafit^®^, Sensus, Roosendaal, The Netherlands), fructooligosaccharides (FOSs; Nutri-Extract, Beroun, Czech Republic), galactooligosaccharides (GOSs; Nutri-Extract, Beroun, Czech Republic), lactulose (Sigma-Aldrich, Saint Louis, MO, USA), raffinose (Sigma-Aldrich, Saint Louis, MO, USA), stachyose (Sigma-Aldrich, Saint Louis, MO, USA), and 2′-fucosyllactose (RAW’s, Metylovice, Czech Republic). A mixture of human milk oligosaccharides (HMOs), which are not available on the market but are commonly found in breast milk, was isolated from human milk according to Rockova et al. [42].

All oligosaccharides were resuspended in Minimum Essential Medium (MEM) with Earle’s Balance salts and L-Glutamine (VWR, CZ) to obtain a final concentration of 10 g/L.

A concentration of 10 g/L was chosen based on typical prebiotic intake (5–10 g/day), which corresponds to an estimated concentration in the colonic lumen assuming an average volume of 0.5–1 L [33,43,44]. Given that the recommended daily intake of beta-glucans is substantially lower (250–500 mg) [45], an additional experiment was performed using a reduced concentration of β-(1,3)-D-glucans at 0.5 g/L. As control groups, cell culture media with glucose (GLU) and cell culture media without added saccharides (WS) were used. The pH values were adjusted to pH 7 for all tested groups. Media were sterilized using syringe microfilters PES (pore size 0.22 μm; Rotilabo^®^, Carl Roth, Karlsruhe, Germany), except for media with beta-(1,3)-D-glucan, which was due to beta-glucan partial solubility in water and high thermostability [46,47], and was sterilized by autoclaving at 121 °C for 15 min. To assess the potential degradation of β-glucans, an amount of released glucose after autoclaving was verified spectrophotometrically using the Glucose Test (Supelco, Bellefonte, PA, USA) and measured with Reflectoquant^®^ RQflex 10 (Merck, Darmstadt, Germany). Post-autoclaving analysis revealed an absence of detectable glucose in the medium [34]. The prepared media were used within 24 h of preparation.

### 2.4. In Vitro Colonic Cell Adhesion Assay

Colonic cell adhesion assay was performed according to previously described methods [27,33,43] with some modifications. Caco-2 cells were seeded in a 96-well culture plate (VWR, CZ) at a density of 2 × 10^5^ cells per well and grown for 14 ± 1 days until confluent at 37 °C in a humidified atmosphere of 5% CO_2_ and 95% air (*v*/*v*). The culture medium was changed every 2 days. Before adding the substrate-supplemented medium and bacterial inoculum, the cell monolayers were washed with PBS to remove antibiotics. Thereafter, the Caco-2 cells were pre-incubated in 37 °C with 90 μL prepared testing media solutions for 20 min. LM inoculums were prepared from fresh overnight cultures grown under aerobic conditions at 37 °C in brain–heart infusion broth (Oxoid, UK), which was checked for purity with a phase-contrast microscope (Nikon Eclipse E200LED MV RS, Nikon, Tokyo, Japan). Bacterial cultures were than centrifuged for 2 min at a speed of 14,500 rpm, washed twice with phosphate-buffered saline (PBS) buffer (VWR, France), and diluted to 5 × 10^8^ cells per 1 mL of the media. These bacterial suspensions were inoculated into each sample to obtain a final density of 5 × 10^7^ CFU/mL [48]. The prepared samples were incubated for 2 h at 37 °C in an atmosphere of 5% [49,50]. All experiments were tested in triplicate. After incubation, the cell layers were washed three times with PBS to remove non-adherent bacteria and finally lysed by the addition of 50 μL of 1% Triton X-100 (Sigma-Aldrich, Prague, Czech Republic) for 30 s. To terminate the lysis process, 150 μL of PBS buffer was added. Bacterial growth was evaluated by the agar plate count technique. Samples were serially ten-fold diluted to a concentration of 10^−7^ and spread on the surface of BHI agar plates. After the incubation at 37 °C for 24 h, colonies were counted and the numbers expressed as colony-forming units (CFU) per 1 mL of the samples. Each of the tested group combinations of LM strains and substrates was analyzed in triplicate. Adhesion data for individual LM strains and substrates were expressed as the percentage of bacteria adhered compared to the total of bacteria added (Musilova et al., 2017 [43]). A general assessment of the impact of individual prebiotics on adherence was evaluated as a percentage of bacteria adhered in the presence of prebiotic compared to the control WS, where the control represents 100% adhesion [30]. Finally, the data was also expressed as logCFU/mL (Table A1).

### 2.5. Imaging of LM and Beta-1,3-D-Glucan Particles

The fresh overnight bacterial suspension was dyed using the fluorescein-5-isothiocyanate (FITC; Life Technologies, Carlsbad, CA, USA) (25 μg/mL) for 30 min at 37 °C in the dark. Bacterial cultures were than centrifuged for 2 min at a speed of 14,500 rpm and washed twice with saline. The prepared bacterial culture was mixed at a 1:10 ratio with the 1% solution of beta-1,3-D-glucan in saline and incubated at 37 °C for 2 h. An effect of beta-1,3-D-glucan particles on LM cells spread was captured immediately after inoculation of the solution (0 h) and after the cultivation (2 h at 37 °C under anaerobic conditions). Imaging was performed a using confocal microscope (ECHO Confocal, BICO, San Diego, CA, USA) with oil objective UPlan X-APO 60X (Olympus^TM^) and ECHO revolution software (version 2.1.8.3) (BICO, USA) using 555 nm fluorescence in a bright field.

### 2.6. Statistical Analysis

The normality of all data sets was tested by Shapiro–Wilk test. The ability of the LM strain to adhere on cell lines (control group WS) was evaluated by the one-way ANOVA followed by Tukey’s post hoc test. The effect of each prebiotic on the adherence of individual LM strain was then evaluated by independent *t*-test. Since the average data of LM adherence capacity did not meet the criterion of normality, the general influence of individual prebiotics on LM adherence was analyzed via Kruskal–Wallis test followed by Tukey’s post hoc test. All the data were processed in Statistica 12 software (StatSoft, Tulsa, OK, USA) with the criterion for significance *p* < 0.05.

## 3. Results

**Figure 1:**
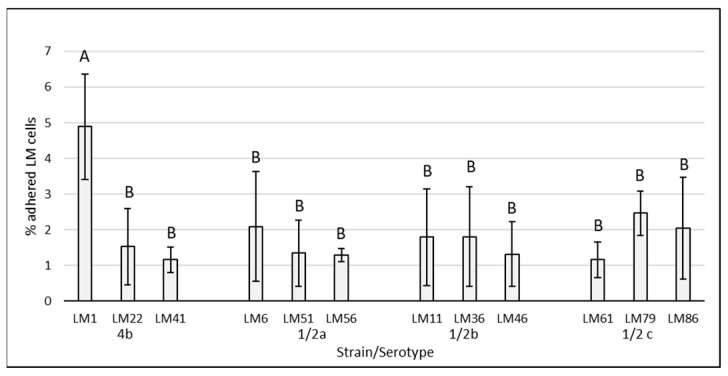
Adhesion of *Listeria monocytogenes* strains of food and food industry origin on Caco-2 cell lines. The data are the means of six independent experiments, expressed as a percentage of the adhered LM compared to the total of bacteria added. The error bars represent the standard deviations. LM: *Listeria monocytogenes*, AB: data with different superscripts differ (*p* < 0.05).

The results of adherence capacity of individual LM strains (without supplementation) are shown in Figure 1. The adhering ability of LM strains ranged from 1.16 ± 0.50% to 4.89 ± 1.48%. The numbers of adhered bacterial cells did not significantly differ among all tested strains, except for LM1 (serotype 4b), which showed statistically significantly higher (*p* < 0.05) adherence.

**Figure 2:**
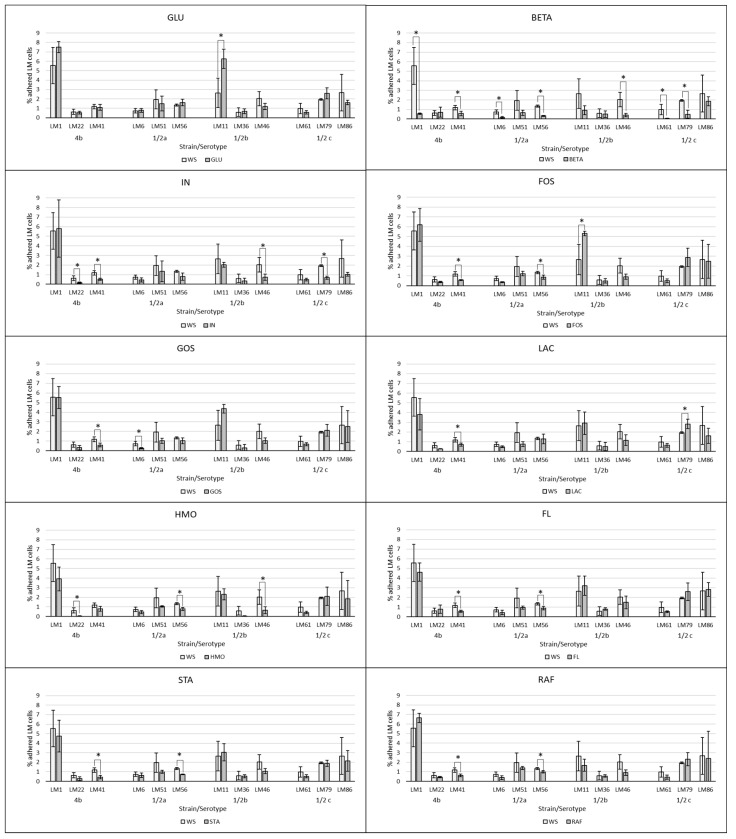
Effect of prebiotics at concentration of 10 g/L on the adherence of individual LM strains compared to the control groups without saccharide supplementation. The data are the means of three independent experiments, expressed as a percentage of the adhered LM compared to the total bacteria added. The error bars represent the standard deviations. WS: without saccharides, GLU: glucose, BETA: beta-(1-3)-D-glucan, IN: inulin, FOS: fructooligosaccharides, GOS: galactooligosaccharides, LAC: lactulose, HMO: mixture of human milk oligosaccharides, FL: 2’-fucosylactose, STA: stachyose, RAF: raffinose, LM: *Listeria monocytogenes*, *: data significantly differ (*p* < 0.05).

**Figure 3:**
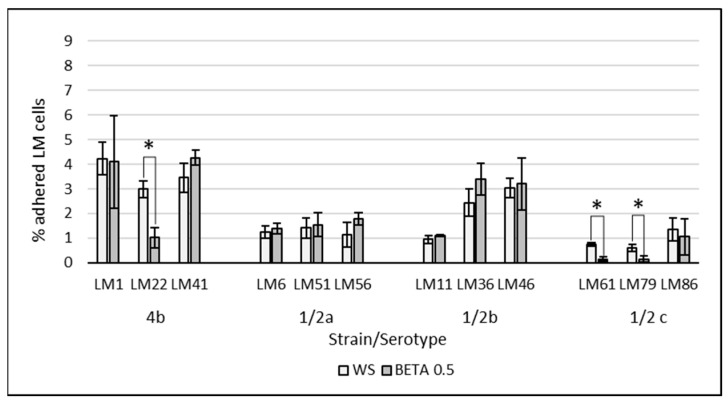
Effect of beta-(1-3)-D-glucan at concentration of 0.5 g/L on the adherence of individual LM strains compared to the control groups without saccharide supplementation. The data are the means of three independent experiments, expressed as a percentage of the adhered LM compared to the total bacteria added. The error bars represent the standard deviations. WS: without saccharides, BETA 0.5: beta-(1-3)-D-glucan at concentration of 0.5 g/L, LM: *Listeria monocytogenes*, *: data significantly differ (*p* < 0.05).

**Figure 4:**
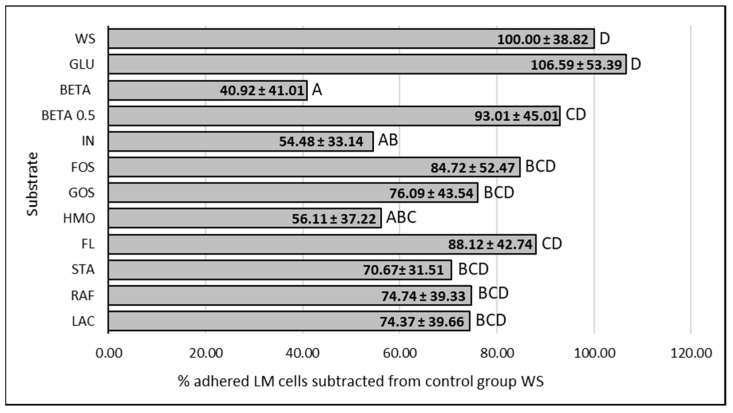
Influence of prebiotics on the average LM adherence ability to Caco-2 cell lines. Data are expressed as percentage of the mean (12 strains, 3 repetitions of each) ± standard deviation of adhered LM in presence of prebiotic in comparation to control without supplemented saccharides, where the control represents 100% adhesion. WS: without saccharides, GLU: glucose, BETA: beta-(1-3)-D-glucan (10 g/L), BETA 0.5: beta-(1-3)-D-glucan (0.5 g/L), IN: inulin, FOS: fructooligosaccharides, GOS: galactooligosaccharides, HMO: mixture of human milk oligosaccharides, FL: 2’-fucosylactose, STA: stachyose, RAF: raffinose, LAC: lactulose, LM: *Listeria monocytogenes*, ABCD: data with different superscripts differ (*p* < 0.05).

The effect of supplemented substrates on the LM adherence is visualized in Figure 2 and Figure 3. The average of all tested LM strains expressed as a % of adhered LM cells subtracted from the control group WS (negative control without saccharides) are visualized in Figure 4. The results of the adherence ability of individual LM strains in numbers of log CFU/mL on Caco-2 cell lines for all treatments are summarized in Table A1. The addition of glucose to the treatment had no significant effect (*p* > 0.05). The only strain that showed a statistically significant (*p* < 0.05) increase in adherence ability after the addition of glucose was strain LM11. Evaluation of the effect of prebiotics on LM adherence revealed that all tested substances were strain-specific and effectively decreased at least one strain. In general, a statistically significant decrease (*p* < 0.05) in LM adherence to Caco-2 cell lines compared to control WS (without saccharides) was observed only for the beta-(1,3)-D-glucan in dosage 10 g/L (adherence inhibited approx. 59%), inulin (adherence inhibited approx. 46%), and HMO treatments (adherence inhibited approx. 44%). Beta-(1,3)-D-glucan significantly (*p* < 0.05) inhibited the adhesion of 7 from 12 tested strains. The reduction for each strain was as follows: LM41: 52 ± 17%, LM6: 77 ± 10%, LM79: 75 ± 21%, LM56: 76 ± 4%, LM46: 79 ± 8%, LM1: 90 ± 1%, LM61: 95 ± 3%). Inulin inhibited the adhesion of four strains (LM46: 64 ± 16%, LM79: 65 ± 9%, LM41: 66 ± 8%, LM22: 73 ± 12%), and HMOs of three strains (LM56: 41 ± 12%, LM46: 67 ± 19%, LM22: 90 ± 3%). Other tested prebiotics overall did not affect (*p* > 0.05) LM adherence ability, similarly to beta-(1,3)-D-glucan in reduced dosage. Beta-(1,3)-D-glucan at the recommended daily dose (0.5 g/L), inhibited LM adherence for three strains (LM22: 65 ± 14%, LM79: 76 ± 19%, LM61: 80 ± 10%).

**Figure 5:**
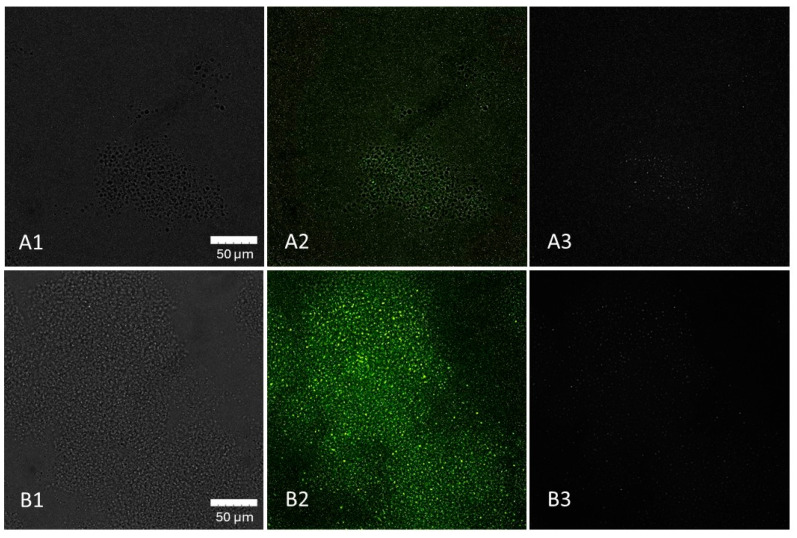
Distribution of *L. monocytogenes* (LM) cells in 1% beta-1,3-D-glucan solution at 0 h (**A1**–**A3**) and after 2 h of incubation (**B1**–**B3**) demonstrated by confocal imaging. (**A1**): beta-1,3-D-glucan particles, (**A2**): beta-1,3-D-glucan with LM cells, (**A3**): LM cells, (**B1**): beta-1,3-D-glucan, (**B2**): beta-1,3-D-glucan with LM cells, (**B3**): LM cells. Captured with an Olympus™ 60X Oil Objective, X-Apo, 1.42NA/0.15WD objective.

Figure 5 illustrates the accumulation of LM cells in the beta-1,3-D-glucan particles. This trend was observed shortly after the inoculation without further incubation.

## 4. Discussion

Prebiotics are non-digestible substrates, mainly non-starch polysaccharides and oligosaccharides, which should be selectively utilized in the intestinal tract by host microorganisms conferring a health benefit [19]. However, in a study by Kodešová [34], it was found that LM strains are able to use beta-(1,3)-D-glucan as a carbon source for their growth. Because the interactions between prebiotics and LM are not yet clearly delineated, this previous study has increased interest in further investigation of other aspects affecting the interactions between prebiotics, LM, and hosts. It is generally acknowledged that prebiotics reduce adherence of pathogenic bacteria, i.e., the risk of infection [22,23,26,33,44,51,52]. Various saccharides can also affect LM metabolism and the gene expression system, including the suppression of pathogenicity, regardless of whether the bacteria are able to metabolize them as an energy source or not [11,13,53,54]. An example is cellobiose, a disaccharide that is metabolizable by LM and simultaneously has a strongly suppressive effect on PrfA expression, the complex virulence gene activator in this bacterium [13,53,54]. The fact that some substances can serve as a carbon source therefore does not necessarily mean an increased risk of disease outbreak arising from their consumption. However, information on the effects of commonly used probiotics on LM adherence capacity is lacking. Based on the above-mentioned facts and the knowledge gap in this field, this study aimed to investigate the effect of selected prebiotics on the adherence capacity of LM strains of food origin to human colorectal adenocarcinoma Caco-2 cell lines.

The adherence of the tested LM strains ranged from 1.16 ± 0.50% to 4.89 ± 1.48%. Our adherence values correspond to those of Laparra et al. and Kushwaha et al. [49,55], who reported the LM adherence ability to Caco-2 cell lines to be approximately 4.09 ± 0.37% [55]. The LM adherence ability was found to be strain-specific. The highest adherence ability was revealed for strain LM1 (serotype 4b). Strain-specificity in bacterial adherence, including *Listeria* spp., is generally known [49,56]. Strain-specificity was also observed in relation of the effect of supplemented substrate, i.e., each strain responded differently depending on the compound. Although Jaradt and Bhunia [57] stated that favorable conditions such as nutrient-rich media and high glucose concentrations (>1.6 g/L) repress listeria adhesive protein (LAP) expression in LM cells, our results showed that glucose had no significant effect on the adhesion. The only exception was the strain LM11 (serotype 1/2b), whose adherence even increased by approximately 100%.

The evaluation of the general effect of individual prebiotics on LM adherence ability showed that beta-(1,3)-D-glucan, inulin, and HMOs were the only substances that statistically significantly reduced (*p* < 0.05) the number of adherent LM cells. Even though the ability of listeria strains to use beta-(1,3)-D-glucan as a nutrient has been noted [34], the greatest reduction in adhered LM cells to Caco 2 cell lines was observed for this prebiotic. In general, the number of adhered bacterial cells (mean of all strains tested) decreased by 59% compared to the control. Beta-(1,3)-D-glucan also significantly affected the adherence of most strains tested (7/12). Reductions ranged from 52% (LM41) to 95% (LM61) depending on the LM strain, which again points to strain-specific characteristics. Several articles describe the immunostimulatory properties and the promotion of host protection against LM by beta-glucans [58,59]. However, to the best of our knowledge, there are no studies evaluating the effect of beta-glucans or structurally similar saccharides on LM adherence to Caco-2 cell lines, with which we could compare our results. The only related finding is that LM is able to adhere to cellulose molecules [9,60,61]. For other substrates (e.g., HMOs, GOSs), a decoy effect, where the pathogen binds to the prebiotic molecules instead of to the binding sites on the intestinal epithelium, has previously been described [25,26]. This issue has not been yet investigated in detail for beta-glucans or beta-glucan-like molecules. However, according to our confocal microscopy results, it is apparent that the bacterial cells accumulated on the beta-1,3-D-glucan particles. However, it cannot be stated with certainty whether the observed interaction represents true bacterial adhesion.

As expected, reducing the concentration of beta-(1,3)-D-glucan to 0.5 g/L resulted in a diminished inhibitory effect on the adherence of most LM strains to Caco-2 cell lines. These findings support the hypothesis that beta-glucans may function through a decoy mechanism, competitively interfering with pathogen binding to host cell receptors. However, given that the adhesion of two strains was only marginally affected and one strain even increased compared to the 10 g/L dose, it must be acknowledged that additional, strain-specific mechanisms may be involved in mediating the interaction between beta-(1,3)-D-glucans and LM adherence, such as, e.g., influence on the expression of adhesins [56,57].

The second prebiotic that most commonly inhibited LM adherence was inulin. Our results showed a statistically significant decrease (*p* < 0.05) in adherent LM cells by approximately 46% on average. However, adherence was affected in only 4/12 strains. The maximum reduction was approximately 70% (LM 22). The effect of inulin on LM adherence has been tested in previous studies, but the results are highly inconsistent. While Ebersbach [27] reported no effect of inulin, Chen et al. [33] reported an increase in LM adherence. In our study, no increase in LM adherence by inulin was found. Differences in results are most likely due to LM strain specificity, or possibly also to differences in culture duration and conditions, or in the methods used to detect adhered cells [49,56,62]. Similarly to beta-glucans, a direct explanation for the reduction in LM adhesion to the Caco-2 line by inulin has not yet been described.

The addition of HMOs to the treatment resulted in an approximately 44% reduction in LM adherence to the Caco-2 cell line in our study. This result is comparable to findings of Chen et al. [33], where adherence was decreased by up to 50%. The maximum adherence decrease was observed for strain LM 22 (serotype), at approximately 90%. However, of the 12 tested strains, only 3 were statistically significantly (*p* < 0.05) inhibited by HMOs. As mentioned above, Shoaf et al. [26] reported that HMOs can act as soluble decoy oligosaccharides for *Campylobacter* and *Vibrio cholera*, ultimately leading to displacement or flushing of the pathogens from the gastrointestinal tract. However, this has not yet been experimentally demonstrated for LM.

For GOSs, FOSs, lactulose, raffinose, stachyose, and 2’-fucosyllactose, no statistically significant (*p* > 0.05) effect on LM adherence was confirmed in general. In some cases, a significant decrease (*p* < 0.05) in individual strains was observed, again confirming strain-specificity. A study by Ebersbach et al. [27], where no effect of GOSs on LM adhesion to the Caco-2 cell line was found either, confirmed that GOSs are unable to significantly affect LM adherence. On the contrary, as mentioned above, Monteagudo-Mera et al. [25] and Shoaf et al. [26] argued that prebiotic oligosaccharides such as GOSs have some structural similarity to cell surface glycoproteins and were postulated to inhibit the adhesion of pathogens to intestinal cells, but this does not seem to be the case for LM. To the best of our knowledge, there are no studies focusing on FOSs, raffinose, stachyose, or 2’-fucosyllactose in this context. In general, research in this area has focused more on interactions between probiotics, prebiotics, and pathogens.

It should also be mentioned that according to results published by Jaradat and Bhunia [56], an in vitro adhesion profile to the Caco-2 cell line might not accurately reflect a strain’s ability to invade cultured cells or organs or tissues in a host model. Additionally, Chen et al. [33] showed that adherence is not the only indicator for evaluating the effects of prebiotics on host health. Other important effects include colonic cell changes or intrinsic cellular responses after prebiotic pretreatment. The resulting outcome depends on the overall mechanisms of action and interactions among prebiotic, pathogen, host microbiome, and host. Cumulative evidence obtained from in vitro and in vivo studies on animal models and human interventions strongly suggest immunostimulatory effects of prebiotics, including beta-(1,3)-glucan [20,58,59]. It should also be added that the regular consumption of prebiotics provides more than just the above-mentioned benefits. Prebiotics are primarily utilized by host commensal bacteria in the intestine, leading to gut microbiota modification by increasing the number of healthy beneficial microorganism species in the gut [43,63,64]. Moreover, in vitro assays showed that, unlike LM, treatment of cell lines with prebiotic saccharides increased the adherence ability of probiotic bacteria to the intestinal epithelium [25,64,65,66]. As a result, the commensal and probiotic bacteria compete for binding sites on the intestinal lining, creating competitive pressure for pathogens. In addition, prebiotics and their metabolites, primarily short-chain fatty acids (SCFAs), which are formed by bacterial fermentation of prebiotics, support the function of GIT epithelial cells and enhance host immunity against pathogens. SCFAs also decrease pH in the digestive tract, which counteracts most pathogens, including LM [19,20,21,22,23,28,67]. Beneficial bacteria such as *Lactobacillus* sp. or some *Bifidobacterium* sp. and commensal bacteria such as *Bacillus* sp., common inhabitants of the digestive tract, utilize different types of prebiotics and produce bacteriocins that inhibit LM. All of these mechanisms also contribute to reducing the risk of LM adherence to the intestinal epithelium [68,69,70]. The finding that prebiotics themselves reduce LM adherence to the intestinal epithelium, even to a small extent, is therefore considered beneficial.

## 5. Conclusions

This study evaluates the effect of various prebiotics on the adherence ability of *Listeria monocytogenes* (LM) to the human adenocarcinoma Caco-2 cell line. The adherence ability was found to be strain-specific, the same as the effect on LM adherence by individual prebiotics. Among the tested compounds, beta-(1,3)-D-glucan, inulin, and human milk oligosaccharides (HMOs) significantly reduced LM adherence. These findings highlight the potential of specific prebiotics to reduce pathogen attachment to the intestinal epithelium and thereby enhance host resistance to LM-associated infections. The observed reduction in adherence is likely mediated by two main mechanisms: (i) a decoy effect, where bacteria bind to prebiotic surfaces instead of host cells, and (ii) modulation of bacterial virulence, particularly the suppression of virulence factors. A lower dose of beta-(1,3)-D-glucan in most cases resulted in a reduced anti-adherence effect on Caco-2 cell lines, which may be due either to a smaller available surface area for bacterial binding or to diminished modulation of bacterial regulatory pathways (i.e., PrfA virulence factor). Both mechanisms or a combination of both may be responsible for this result. These findings indicate an interaction between beta-glucans and *Listeria* spp.; therefore, further research exploring additional molecular mechanisms could be considered.

## Figures and Tables

**Table 1:** Tested *Listeria monocytogenes* (LM) strains.

Sample	Accession No.	Serotype	Origin
LM1	OR725603	4b	salami (Vysočina)
LM22	OR725606	4b	sausages (Spišské)
LM41	OR725608	4b	raw beef meat (steak tartare)
LM11	OR725605	1/2b	smear of a work surface within the food industry
LM36	OR725607	1/2b	tee sausage
LM46	OR725609	1/2b	smear of a work surface within the food industry
LM6	OR725604	1/2a	cheese-stuffed pickled peppers in oil
LM51	OR725610	1/2a	vegetarian burger
LM56	OR725611	1/2a	raw beef meat (steak tartare)
LM61	OR725612	1/2c	raw minced and mixed beef and pork meat
LM79	OR725613	1/2c	vegetarian minced meat
LM86	OR725614	1/2c	raw pork meat (steak tartare, Cibuláček)

## Data Availability

The original contributions presented in this study are included in this article/Supplementary Materials; further inquiries can be directed to the corresponding authors.

## Supplementary Materials

The following supporting information can be downloaded at https://www.mdpi.com/article/10.3390/cimb47110891/s1.

## Appendix A

**Table A1:** Adhesion of individual *Listeria monocytogenes* strains adhered to Caco-2 cell lines in various treatments expressed as log CFU/mL ± SD.

Strain	Serotype	BETA	IN	GOS	FOS	LAC	RAF	STA	2’FL	HMO	GLU	WS
LM1 *	4b	5.45 ± 0.06 ^b^	6.43 ± 0.22 ^a^	6.44 ± 0.08 ^a^	6.48 ± 0.13 ^a^	6.25 ± 0.19 ^a^	6.52 ± 0.03 ^a^	6.36 ± 0.17 ^a^	6.36 ± 0.08 ^a^	6.28 ± 0.15 ^a^	6.57 ± 0.03 ^a^	6.43 ± 0.13 ^aA^
LM22		5.45 ± 0.36 ^ab^	4.91 ± 0.17 ^ab^	5.13 ± 0.32 ^ab^	5.26 ± 0.08 ^ab^	5.12 ± 0.03 ^ab^	5.34 ± 0.07 ^ab^	5.09 ± 0.28 ^ab^	5.54 ± 0.30 ^a^	4.49 ± 0.13 ^b^	5.41 ± 0.13 ^ab^	5.47 ± 0.18 ^abB^
LM41		5.44 ± 0.15 ^bc^	5.41 ± 0.08 ^c^	5.46 ± 0.14 ^bc^	5.45 ± 0.05 ^bc^	5.54 ± 0.09 ^abc^	5.48 ± 0.11 ^bc^	5.33 ± 0.18 ^c^	5.45 ± 0.10 ^bc^	5.58 ± 0.14 ^abc^	5.73 ± 0.12 ^ab^	5.77 ± 0.09 ^aB^
LM6	1/2a	4.90 ± 0.19 ^c^	5.32 ± 0.20 ^abc^	5.12 ± 0.14 ^bc^	5.26 ± 0.07 ^abc^	5.38 ± 0.08 ^abc^	5.26 ± 0.22 ^a^	5.47 ± 0.19 ^abc^	5.28 ± 0.30 ^abc^	5.35 ± 0.16 ^abc^	5.59 ± 0.12 ^a^	5.55 ± 0.16 ^abB^
LM51		5.48 ± 0.20 ^a^	5.74 ± 0.36 ^a^	5.70 ± 0.13 ^a^	5.78 ± 0.08 ^a^	5.56 ± 0.16 ^a^	5.84 ± 0.05 ^a^	5.69 ± 0.07 ^a^	5.68 ± 0.07 ^a^	5.72 ± 0.03 ^a^	5.82 ± 0.28 ^a^	5.94 ± 0.24 ^aB^
LM56 *		5.19 ± 0.07 ^c^	5.56 ± 0.22 ^bc^	5.70 ± 0.12 ^abc^	5.63 ± 0.01 ^abc^	5.79 ± 0.18 ^ab^	5.69 ± 0.06 ^abc^	5.56 ± 0.03 ^bc^	5.65 ± 0.09 ^abc^	5.59 ± 0.10 ^bc^	5.90 ± 0.10 ^a^	5.83 ± 0.20 ^abB^
LM11 *	1/2b	5.63 ± 0.22 ^d^	6.00 ± 0.05 ^cd^	6.34 ± 0.04 ^abc^	6.42 ± 0.02 ^ab^	6.14 ± 0.19 ^bcd^	5.90 ± 0.16 ^d^	6.17 ± 0.14 ^bcd^	6.19 ± 0.14 ^bcd^	6.05 ± 0.11 ^cd^	6.49 ± 0.08 ^a^	6.07 ± 0.24 ^cdAB^
LM36		5.30 ± 0.39 ^a^	5.06 ± 0.42 ^a^	5.03 ± 0.46 ^a^	5.35 ± 0.26 ^a^	5.29 ± 0.36 ^a^	5.43 ± 0.10 ^a^	5.41 ± 0.13 ^a^	5.60 ± 0.06 ^a^	4.32 ± 0.32 ^a^	5.51 ± 0.19 ^a^	5.39 ± 0.32 ^aB^
LM46		5.29 ± 0.22 ^b^	5.54 ± 0.18 ^b^	5.71 ± 0.12 ^ab^	5.64 ± 0.14 ^ab^	5.72 ± 0.24 ^ab^	5.63 ± 0.16 ^ab^	5.72 ± 0.11 ^ab^	5.84 ± 0.19 ^ab^	5.47 ± 0.26 ^b^	5.76 ± 0.12 ^ab^	5.98 ± 0.19 ^aB^
LM61	1/2c	4.29 ± 0.33 ^b^	5.35 ± 0.15 ^ab^	5.52 ± 0.11 ^ab^	5.37 ± 0.18 ^ab^	5.48 ± 0.16 ^ab^	5.32 ± 0.21 ^ab^	5.40 ± 0.16 ^ab^	5.42 ± 0.08 ^ab^	5.31 ± 0.14 ^ab^	5.43 ± 0.16 ^ab^	5.63 ± 0.32 ^aB^
LM79 *		5.29 ± 0.34 ^b^	5.52 ± 0.11 ^b^	6.01 ± 0.12 ^ab^	6.14 ± 0.15 ^a^	6.15 ± 0.08 ^a^	6.05 ± 0.15 ^ab^	5.97 ± 0.08 ^ab^	6.09 ± 0.15 ^a^	6.00 ± 0.18 ^ab^	6.10 ± 0.10 ^a^	5.99 ± 0.02 ^abB^
LM86		5.96 ± 0.11 ^a^	5.71 ± 0.09 ^a^	6.04 ± 0.29 ^a^	6.00 ± 0.39 ^a^	5.87 ± 0.24 ^a^	5.87 ± 0.51 ^a^	5.98 ± 0.26 ^a^	6.14 ± 0.11 ^a^	5.77 ± 0.56 ^a^	5.90 ± 0.07 ^a^	6.00 ± 0.46 ^aAB^

All data for individual strains are means from triplicate determination ± SD. The data were evaluated by the one-way ANOVA followed by Tukey’s post hoc test. BETA: beta-(1-3)-D-glucan, IN: inulin, GOS: galactooligosaccharides, FOS: fructooligosaccharides, LAC: lactulose, RAF: raffinose, STA: stachyose, HMO: mixture of human milk oligosaccharides, 2’FL: 2’-fucosylactose, GLU: control group with glucose, WS: control group without saccharides, SD: standard deviation. * LM strains tested for the ability to utilize prebiotics (Kodešová et al., 2023 [34]). ^abcd^ data with different superscripts differ (*p* < 0.05) inline. ^AB^ data with different superscripts differ (*p* < 0.05) in a column. Evaluated only for control group WS.
