# Supplementary material for: Effect of Different Prebiotic Saccharides on Listeria monocytogenes Adherence to Human Adenocarcinoma Caco-2 Cell Line
Source: Curr Issues Mol Biol. 2025 Oct 28;47(11):891. doi: 10.3390/cimb47110891 (PMC12650974; doi:10.3390/cimb47110891)
Supplement: Supplementary file 1 [file cimb-47-00891-s001.zip › cimb-3904669-supplementary.pdf]

Table S1. Sequences of primers used to detect genes encoding *Listeria* adhesion proteins, cycling condition during PCR, and resultant amplicon size.

Adhesion-associated proteins	Gene	Primer	Sequence (5′–3′)	Amplicon length (bp)	PCR conditions			References
					°C	Time	Cycles	
					94	5 min		
		inlB F	CATGGGAGAGTAACCCAACC		94	30 sec		
Internalin B	*InlB*			500	57	30 s	30	Zhang et al., 2005 [1]
		inlB R	GCGGTAACCCCTTTGTCATA		72	30 s		
					72	10 min		
Internalin A	*InlA*	inlA-F	ACGAGTAACGGGACAAATGC	800	94	2 min		
		inlA-R	CCCGACAGTGGTGCTAGATT		94	20 sec		
Internalin C	*InlC*	inlC-F	AATTCCCACAGGACACAACC	517	55	20 sec	30	Liu et al., 2007 [2]
		inlC-R	CGGGAATGCAATTTTTCACTA		72	50 sec		
Internalin J	*nlJ*	inlJ-F	TGTAACCCCGCTTACACAGTT	238	72	2 min		
		inlJ-R	AGCGGCTTGGCAGTCTAATA					
					94	5 min		
		LAP-F	TCCTCACGGTCGTGCCAATGC		94	30 sec		
Liasteria adhesion protein	*LAP*			157	60	1 min	30	Ebersbach et al., 2012 [3]
		LAP-R	AGTTGCAGCAGGGAAGCCGA		72	30 sec		
					72	2 min		
					94	5 min		
		iapF	ACAAGCTGCACCTGTTGCAG		94	30 sec		
Invasion associated protein	*iap*			131	55	1 min	30	Furrer et al., 1991* [4]
		iapR	TGACAGCGTGTGTAGTAGCA		72	1 min		
					72	2 min		

*Presumptive β-hemolysin gene in reference

Standard PCRs were conducted in a volume of 25 µl containing 2 µl of DNA, 1 µL of each primer (10 µM), 12.5 µL DreamTaq Green PCR Master Mix (Thermo Fisher), and 8.5 µL of nuclease-free water (Thermo Fisher). Multiplex PCR for simultaneous detection of genes encoding internalin A, internalin C, and internalin J was performed in a volume of 25 µl containing 2 µl of DNA, 2 µL of each inlA primer (10 µM), 1,5 µL of each inlC primer, 1 µL of each inlJ primer 12.5 µL DreamTaq Green PCR Master Mix (Thermo Fisher), and 2.5 µL of nuclease-free water. (Thermo Fisher) in an automated T100 Thermal cycler (Bio-Rad). Amplification reactions were carried out in an automated T100 Thermal cycler (Bio-Rad). The PCR products were electrophoresed in a 1% agarose gel stained with GelRed™ (Biotium) for 60 min at a constant voltage of 130 V in 1× TAE buffer (40 mM Tris, 20 mM acetic acid, 1 mM EDTA, Fermentas). GeneRuler DNA Ladder Mix (Thermo Fisher) was loaded into the gel as a molecular marker, together with samples. The PCR bands were then visualized under a UV transilluminator (Bio-Rad).

References:

1. Zhang, W.; Knabel, S.J. Multiplex PCR Assay Simplifies Serotyping and Sequence Typing of Listeria Monocytogenes Associated with Human Outbreaks. *J Food Prot* 2005, *68*, 1907–1910, doi:10.4315/0362-028X-68.9.1907.

2. Liu, D.; Lawrence, M.L.; Austin, F.W.; Ainsworth, A.J. A Multiplex PCR for Species- and Virulence-Specific Determination of Listeria Monocytogenes. *J Microbiol Methods* 2007, *71*, 133–140, doi:10.1016/J.MIMET.2007.08.007.

3. Ebersbach, T.; Andersen, J.B.; Bergström, A.; Hutkins, R.W.; Licht, T.R. Xylo-Oligosaccharides Inhibit Pathogen Adhesion to Enterocytes in Vitro. *Res Microbiol* 2012, *163*, 22–27, doi:10.1016/J.RESMIC.2011.10.003.

4. Furrer, B.; Candrian, U.; Hoefelein, C.; Luethy, J. Detection and Identification of Listeria Monocytogenes in Cooked Sausage Products and in Milk by in Vitro Amplification of Haemolysin Gene Fragments. *Journal of Applied Bacteriology* 1991, *70*, 372–379, doi:10.1111/J.1365-2672.1991.TB02951.X.
